# FUTURE CARE PLANNING AND END-OF-LIFE CARE DECISION MAKING: INDIVIDUAL AND SOCIAL INFLUENCES

**DOI:** 10.1093/geroni/igz038.1586

**Published:** 2019-11-08

**Authors:** Jeong Eun Lee, Silvia Sörensen

**Affiliations:** 1 Iowa State University, Ames, Iowa, United States; 2 University of Rochester Warner School for Education and Human Development, Rochester NY, United States

## Abstract

As a growing number of older adults reach very old age, future care planning and end-of-life care decision making becomes increasingly important. Previous studies have shown that concrete future care planning steps are related to improved ability to manage illness and to better mental and physical health outcomes among older adults. Yet, relatively few older adults sufficiently plan for their future care. The purpose of this symposium is to highlight a collection of studies that each brings a unique perspective to the issue, reporting on individual and social factors that influence future care planning, end-of-life care decision making, and strategies to enhance future care planning among older adults. First, Chen and Siconolfi address common barriers and facilitators across diverse domains of age-related planning using content analysis. Second, Boerner and colleagues focus on the completion of formal planning without discussing the contents and factors associated with formal planning completion. Third, Strum investigates the complexities of navigating “fair” later life decisions involving family resources. Fourth, Moorman examines the racial differences in decisions of euthanasia and physician assisted suicide. Finally, Lee and colleagues report the findings from a future care planning intervention with older adults in rural area. The discussion by Sörensen will integrate the five papers with the goal of connecting the current evidence for meaningful steps in research and practice related to future care planning in older populations.

